# Non-alcoholic beverages and risk of bladder cancer in Uruguay

**DOI:** 10.1186/1471-2407-7-57

**Published:** 2007-03-29

**Authors:** Eduardo De Stefani, Paolo Boffetta, Hugo Deneo-Pellegrini, Pelayo Correa, Alvaro L Ronco, Paul Brennan, Gilles Ferro, Giselle Acosta, María Mendilaharsu

**Affiliations:** 1 Grupo de Epidemiología. Departamento de Anatomía Patológica, Hospital de Clínicas, Facultad de Medicina, Montevideo, Uruguay; 2 International Agency for Research on Cancer, Lyon, France; 3 Department of Pathology, Louisiana State University Health Sciences, New Orleans, Louisiana, USA; 4 Departamento de Epidemiología, Sección de Radiología, Hospital Pereira Rossell, Montevideo, Uruguay

## Abstract

**Background:**

Bladder cancer is the fourth most frequent malignancy among Uruguayan men. A previous study from Uruguay suggested a high risk of bladder cancer associated with *maté *drinking. We conducted an additional case-control study in order to further explore the role of non-alcoholic beverages in bladder carcinogenesis.

**Methods:**

In the time period 1996–2000, 255 incident cases with transitional cell carcinoma of the bladder and 501 patients treated in the same hospitals and in the same time period were frequency matched on age, sex, and residence. Both cases and controls were face-to-face interviewed on occupation, tobacco smoking, alcohol drinking and intake of *maté*, coffee, tea, and soft drinks. Statistical analysis was carried out by unconditional multiple logistic regression.

**Results:**

*Ever maté *drinking was positively associated with bladder cancer (odds ratio [OR] 2.2, 95% confidence interval [CI] 1.2–3.9) and the risk increased for increasing duration and amount of *maté *drinking. Both coffee and tea were strongly associated with bladder cancer risk (OR for coffee drinking 1.6, 95% CI 1.2–2.3; OR for tea drinking 2.3, 95% CI 1.5–3.4). These results were confirmed in a separate analysis of never-smokers.

**Conclusion:**

Our results suggest that drinking of *maté*, coffee and tea may be risk factors for bladder carcinoma in Uruguay.

## Background

Bladder cancer is the fourth most frequent malignancy among Urugan men, with age-standardized incidence rate of 19.7 per 100,000 [1]. In international comparisons between registries of the Americas, incidence among Uruguayan men is second only to that among White men in the United States [1].

A previous study from Uruguay reported an increased risk of bladder cancer associated with *maté *drinking, a local herbal tea derived from the plant known as *Ilex paraguariensis *[2]. This non-alcoholic beverage has been considered as a risk factor for esophageal cancer [7,8]. According to the International Agency for Research on Cancer *maté *drinking is a probable carcinogen to humans (Group 2A) possibly acting through thermal injury on esophageal mucosa [9]. In effect, *maté *is drunk usually very hot, but the effect of *maté *drinking on the bladder mucosa cannot be explained by temperature. It is possible that *maté *could contain some carcinogens, not yet discovered. On the other hand, since *maté *drinking is correlated with tobacco smoking, it is difficult to discard residual confounding by smoking.

Also coffee and tea drinking has been suspected as bladder carcinogens, but the current evidence of such effect is controversial [9]. For this reason we have decided to conduct a case-control study on the role of non-alcoholic beverages in the etiology of bladder cancer in the population of Uruguay characterized by high rates of bladder cancer.

## Methods

### Selection of cases

In the time period 1996–2000, 261 newly diagnosed and microscopically confirmed consecutive cases of patients afflicted by transitional cell carcinoma of the bladder were identified in the four major hospitals of Montevideo (Cancer Institute, Pasteur, Clinicas and Maciel). Six patients refused the interview, leaving a final total of 255 cases (response rate 97.7 %). Most patients were males (225 patients) and there were only 30 females. All cases were interviewed within one month of their diagnosis.

### Selection of controls

In the same time period and in the same hospitals, seven hundred patients with diseases not related with tobacco smoking, alcohol drinking and without recent changes in their diet were considered eligible for this study. Thirty-one patients refused the interview, leaving a final total of 669 potential controls (response rate 95.6 %). Five hundred and one patients were included as controls in the present study. They were randomly selected from amongst those fulfilling the matching criteria (age [5-year groups], sex, residence [Montevideo, other counties]. It is important to emphasize that cases and controls do not overlap with those included in the previous Uruguayan study on bladder cancer and *maté *drinking [2]. On the other hand, there was some overlapping with a second study on bladder cancer conducted in Uruguay [3]. The controls presented the following diseases: abdominal hernia (120 patients, 23.8 %), eye disorders (100, 20.0 %), diseases of the skin (58, 11.6 %), urinary stones (41, 8.2 %), acute appendicitis (38, 7.6 %), injuries (38, 7.6 %), varicose veins (32, 6.4 %), hydatid cyst (27, 5.4 %), prostate hypertrophy (25, 5.0 %) and blood disorders (22, 4.4 %).

### Questionnaire

All participants (controls and cases) were face-to-face interviewed in the hospitals by two trained social workers. They administered a structured questionnaire with following sections: sociodemographics, a complete occupational history based in job titles, self-reported height and weight 5 years before the date of the interview, family history of cancer among first-degree relatives, a complete history of tobacco smoking including smoking status, number of cigarettes smoked per day, years of smoking, type of tobacco and type of cigarette, a complete history of alcohol drinking including age at start, years since quitting, number of glasses drunk per day or week, a complete history of *maté *drinking including age at start, years since quitting, liters or fractions of liters drunk per day, and usual temperature (warm, hot, very hot, based on results of a validation study conducted in Southern Brazil [10]), a complete history of coffee drinking, and a complete history of tea drinking. The questionnaire on fluid intake was based on that used in the previous study in Uruguay [2]. In addition, we used a food frequency questionnaire (FFQ) which included 64 queries on frequency of consumption of common foodstuffs five years before interview. The FFQ was designed in order to cover the usual Uruguayan diet and allowed the calculation of total energy intake: it was tested for reproducibility with good results (results available). For the beverages included in the present analysis (*maté*, coffee, tea and soft drinks [e.g., Coca-Cola, Pepsi-Cola, Sprite]), indicators of frequency of intake (cups or liters per week) and of cumulative intake (frequency indicators multiplied by duration of intake, in the form of cup-years or liter-years) were used.

### Definition of high-risk occupations

The following occupations were considered high-risk jobs: dyestuff workers, dye users, rubber workers, leather workers, painters, truck drivers, metal workers, printers, textiles, butchers, construction workers and roofers, based on results of preliminary analyses of occupational risk factors of bladder cancer in this study population.

### Statistical analysis

Relative risks, approximated by the odds ratios (OR's) and corresponding 95 percent confidence intervals (95 % CI's) were estimated by unconditional multiple logistic regression [11]. The basic model included the following terms: age (categorical, 6 strata), sex, residence (categorical), urban/rural status (categorical), education (categorical, 3 strata), family history of bladder cancer among first degree relatives (no/yes), body mass index (categorical), employment in high-risk occupations (no/yes) and tobacco smoking (smoking status, number of cigarettes smoked per day, years since quitting and age at start smoking), *maté *drinking (categorical), coffee drinking (categorical), tea drinking (categorical) and milk intake (categorical). The analyses were repeated after stratification for smoking status. Tests for linear trend were performed after entering categorical variables as ordinal (continuous) in the same model. Departure from the multiplicative model was determined by assessing the likelihood ratio test statistic. An alpha level of 0.05 was used as the indicator of statistical significance. All p-values were derived from two-sided statistical tests. All the calculations were done with the STATA programme [12].

The study was approved by the ethics committee of the International Agency for Research on Cancer. Participants signed an informed consent form to participate in the study.

## Results

The distribution of cases and controls by sociodemographic variables, potential confounders and tobacco smoking is shown in Table 1. As a result of the matched design, the distribution by age and sex were similar, while a higher proportion of cases than controls lived outside Montevideo. The proportion of rural cases was significantly higher compared with controls, and cases were more educated and earned higher incomes than controls. There was a significantly higher proportion of cases with family history of bladder cancer compared with controls (OR 5.0, 95 % CI 1.7–4.3). Both series of patients displayed similar BMI. Cases worked more frequently in high-risk occupations compared with controls (OR 1.4, 95 % CI 0.9–2.1). Finally, cases were more frequently smokers compared with controls (OR 1.9, 95 % CI 1.2–2.9).

Odds ratios of bladder cancer for *maté *drinking are shown in Table 2. Former drinkers displayed higher risks compared with current drinkers. It is important to note that only 13 cases and 17 controls were ex-drinkers. Ever (i.e., current or former) drinkers of *maté *experienced an OR of 2.2 (95 % CI 1.2–3.9). Amount of *maté *drunk per week was positively associated with bladder cancer risk (OR for heavy drinkers 3.7, 95 % CI 1.9–7.1, p-value for trend < 0.01). There was also a dose-response relationship between years of drinking *maté *and bladder cancer risk (OR for long-term drinkers 3.0, 95 % CI 1.5–6.0, p-value for trend < 0.01). Cumulative exposure to *maté*, measured in liter-years, was directly associated with a three-fold increase in risk and those exposed to very hot *maté *displayed an OR of 4.9 (95 % CI 2.2–11) (reference category: never drinkers).

Odds ratios of bladder carcinoma for consumption of coffee, tea and soft drinks are shown in Table 3. Coffee consumption was directly associated with bladder cancer risk. This applies to former and current drinkers of both pure coffee and coffee with milk. Although drinking of coffee with milk was apparently associated with a higher risk of bladder cancer than drinking of black coffee, the difference was not statistically significant. Also, tea drinking was directly associated with risk of bladder cancer. The increase in risk was similar for amount of pure tea and for tea with milk (OR 6.5, 95 % CI 2.0–21, p-value for trend = 0.002). Finally, drinking of soft drinks was not associated with risk of bladder cancer.

Coffee drinking was positively correlated with tea drinking (coefficient 0.09, p = 0.02), while *maté *drinking was negatively correlated with drinking of coffee (coefficient -0.07, p = 0.07) and tea (-0.11, p = 0.002).

Table 4 presents results on drinking of *maté*, coffee and tea separately for ever- and never-smokers. In the case of coffee drinking, the results were similar in the two groups, although the precision of risk estimates in never smokers was reduced by the relatively small number of subjects. In the case of drinking of *maté *and tea, an effect was suggested also in never smokers, although of lesser magnitude than among smokers. Overall these results suggest that the increased risk of bladder cancer for intake of non-alcoholic beverages do not appear to be completely explained by smoking.

## Discussion

The most important finding of our study was the significant increase in risk of bladder cancer associated with *maté *consumption. Most previous studies on *maté *drinking and human cancer [7-9,13-15] were conducted in cancer sites on which *maté *ingestion may be in direct contact with the epithelium. This fact reinforces the hypothesis that *maté *drinking acts by thermal injury. A complementary mechanistic hypothesis is that *maté *could contain chemical carcinogens. An unpublished chemical analysis (R.D. Adams and D. Hoffmann, personal communication) failed to obtain evidences of the presence of N-Nitroso compounds. On the other hand, one study reported the presence of large amounts of benzo [a]pyrene in eight commercial samples of *maté *leaf bought in Germany; however, the beverage resulting from infusion of the leaves contained only 0.02–0.12 micrograms/liter [16]. Other chemical studies suggested that *maté *could have carcinogenic effects due to its contents in tannins [17,18]. Experimental studies in rats and mice showed that caffeic acid (a metabolite of cholorogenic acid which is abundant in *maté *leaves) has carcinogenic effects on the kidney [19]. Experimental studies in Brazil suggested that *maté *displayed mutagenic and clastogenic activities in cell cultures [20,21]. Finally, in an experimental study in animals, Roffo [22] was able to produce squamous cell carcinomas of the skin after painting this organ with "tar" from *maté*. Recently, Fagundes and colleagues (personal communication) studied the urine in *maté *drinkers and found evidences of high levels of PAH derived from tobacco and *maté*.

Since there exist the possibility that *maté *could contain chemicals with carcinogenic activity, it was suggested that epidemiologic studies on *maté *drinking and cancer sites not related with *maté *temperature in humans could be a useful model to test this possibility. Up to date five such studies were conducted. Three case-control studies conducted in Uruguay [2,23,24] displayed significant positive associations between *maté *drinking and lung, renal cell and bladder cancers. One additional case-control from Argentina study was characterized by a strong direct association between coffee consumption and bladder cancer, but no effect of *maté *drinking [25]. Finally, in a recent case-control study conducted in Córdoba, Argentina, *maté *was associated with bladder cancer risk [26]. No data were previously reported on effect of temperature of *maté *on bladder cancer risk.

Thus, it remains the strong possibility that *maté *drinking could be carcinogenic to cancer sites not related with a direct contact with the beverage. In other words, our findings of a strong direct association between *maté *drinking and bladder cancer risk are partially supported by previous chemical, experimental and epidemiological studies in cancer of the bladder and of other organs.

Another possibility is related with residual confounding of *maté *drinking by tobacco smoking. In the present study, the analysis of *maté *drinking was adjusted for smoking status, cigarettes per day and years since quit. Moreover, since there were a sizeable number of never smokers, this allowed to estimate the effect of *maté *drinking among this subset. Although results among never smokers were less precise than those obtained in the whole study population, the increased risk for heavy drinking of *maté *obtained in this subgroup detracts from the hypothesis of confounding by tobacco smoking.

Concerning the effect of other non-alcoholic beverages, both coffee and tea drinking were directly associated with bladder cancer risk, mainly when coffee is ingested with milk. The latter result was unexpected and can be due to chance or residual confounding. It might also indicate the presence of bladder carcinogens in black tea. Previous studies on tea intake and bladder cancer risk yielded controversial results. At least one prospective study [27] and two case-control studies [28,29] reported significant increases in risk of bladder cancer, whereas the remaining studies failed to show an association between tea consumption and bladder cancer risk [30]. Thus, there is no consistent evidence of a carcinogenic effect of tea on bladder mucosa. The possibility of residual confounding from smoking is very difficult to exclude since the OR's for tea drinking among never smokers was not clearly elevated, although the number of tea drinkers was rather small. Chance remains an additional possible explanation of these findings.

We did not ask about intake of water. However, this would create a bias only if drinking of water were associated with bladder cancer risk and is correlated to drinking of the beverages under study. Indeed, low overall fluid intake (represented mainly by water) has been suggested as a possible risk factor for bladder cancer [31], but this would act as a positive confounder in our study only if high intake of *maté*, coffee or tea would associated with low overall fluid intake. The lack of information on source of drinking water and potential exposure to drinking water contaminants such as chlorination by-products and nitrates is an additional limitation of our study.

The present study, as other hospital-based case-control studies, is subjected to several limitations. Selection bias is almost impossible to rule out. We tried to minimize this bias by frequency matching controls and cases on age, sex and residence. Although matching for the latter variable was not complete, we adjusted for it in all analyses. Furthermore, we have adjusted the risk estimates for possible determinants of selection of cases and controls, such as urban/rural status and education.

Misclassification bias is also difficult to exclude. It is important to note that the role of nonalcoholic beverages in bladder cancer risk is unknown, both by the general population, the hospital population and the interviewers. Thus, is rather unlikely that differential or nondifferential exposure to *maté *drinking has occurred in our study. Furthermore, although current intake of non-alcoholic beverages among controls might have been modified by their disease, we consider unlikely that the use of hospital controls has created a bias in the prevalence of lifetime exposure. A further limitation is the relatively small sample size of the study, which reduced the power of detecting interactions between risk factors (e.g., differences in *maté*-related OR according to smoking status) and differences according to subtle differences in exposure (e.g., whether the effect of coffee with milk is really different from that of black coffee, as suggested by our results). On the other hand our study has strengths. Perhaps the more important strength is related with the high response rate in both series of patients (cases and controls). Another strength is the absence of proxy interviews. Finally, the sizeable number of never smokers, which allowed to estimate OR's of *maté *drinking among these subset of patients, is a strength of the study.

## Conclusion

The present study replicates previous findings from Uruguay suggesting a role of *maté *in bladder carcinogenesis. Also, the effect of *maté *drinking among never smokers suggest that this beverage is an independent factor. Future studies should confirm or reject the hypothesis of a role of *maté *in bladder carcinoma. If these findings are confirmed, *maté *drinking is likely to contribute to the high risk of bladder cancer in Uruguay and other South American countries in which the habit is widespread.

## Competing interests

The author(s) declare that they have no competing interests.

## Authors' contributions

EDS, PB, PC and PBr designed the study; EDS, HDP, ALR, GA and MM collected the data; EDS, PB and GF analyzed the data; EDS and PB drafted the manuscript. All authors reviewed and approved the manuscript.

## Pre-publication history

The pre-publication history for this paper can be accessed here:



## Figures and Tables

**Table 1:** Distribution of controls and cases by sociodemographic variables and selected risk factors

		**Cases**		**Controls**		**OR**	**95% CI**
**Variable**	**Category**	**Number**	**%**	**Number**	**%**		

Age (years)	30–39	2	0.8	4	0.8	NA	
	40–49	14	5.5	28	5.6		
	50–59	37	14.5	74	14.8		
	60–69	91	35.7	182	36.3		
	70–79	86	33.7	175	34.9		
	80–89	25	9.8	38	7.6		
Sex	Males	225	88.2	441	88.0	NA	
	Females	30	11.8	60	12.0		
Residence	Montevideo	116	45.5	254	50.7	NA	
	Other counties	139	54.5	247	49.3		
Urban/rural status	Urban	192	75.3	416	83.0	1.00	
	Rural	63	24.7	85	17.0	1.31	0.80–2.14
Education (years)	0–2	57	22.3	140	27.9	1.00	
	3–5	106	41.6	175	34.9	1.42	0.93–2.17
	6+	92	36.1	186	37.2	1.24	0.79–1.95
Income (US dollars per month)	<=142	93	36.5	225	44.9	1.00	
	143+	113	44.3	194	38.7	1.51	1.05–2.17
	Missing	49	19.2	82	16.4	1.50	0.94–2.40
Family history of bladder cancer	No	244	95.7	495	98.8	1.00	
	Yes	11	4.3	6	1.2	4.97	1.73–14.27
Body Mass Index	<=22.8	59	23.1	127	25.3	1.00	
	22.9–25.1	65	25.5	126	25.1	1.13	0.72–1.79
	25.2–27.1	53	20.8	120	23.9	0.92	0.57–1.49
	27.2+	78	30.6	128	25.7	1.33	0.84–2.10
Occupation	Low risk	175	68.6	385	76.9	1.00	
	High risk	80	31.4	116	23.1	1.41	0.92–2.15
Years of smoking	Never smokers	48	18.8	136	27.1	1.0	Reference
	1–29	32	12.5	60	12.0	1.55	0.85–2.82
	30–39	31	12.2	70	14.0	1.34	0.73–2.46
	40–49	54	21.2	109	21.8	1.61	0.94–2.74
	50+	90	35.3	126	25.1	2.39	1.46–3.90
N patients		255	100.0	501	100.0		

NA, not applicable (matching variable)

OR, odds ratio adjusted for age, sex, residence, urban/rural status, education, family history of bladder cancer among first-degree relatives, body mass index, occupation, smoking status, years since quit, number of cigarettes smoked per day, *maté *drinking, coffee drinking, tea drinking, soft drinks and milk intake

CI, confidence interval

**Table 2:** Odds ratios of bladder cancer for *maté *drinking

	**Category**	**Cases/Controls**	**OR**	**95 % CI**
*Maté *drinking	Never drinkers	18/71	1.0	
	Former drinkers	13/17	3.1	1.2–8.1
	Current drinkers	224/413	2.2	1.2–3.9
	Ever drinkers	237/430	2.2	1.2–3.9
Amount of drinking	Never drinkers	18/71	1.0	
(liters/day)	0.1–0.9	31/89	1.3	0.6–2.7
	1.0–1.9	125/248	2.1	1.2–3.9
	2.0+	81/93	3.7	1.9–7.1
	p-value for trend		<0.01	
Duration of drinking	Never drinkers	18/71	1.0	
(years)	1–49	84/180	1.7	0.9–3.3
	50–59	85/155	2.4	1.3–4.5
	60+	68/95	3.0	1.5–6.0
	p-value for trend		<0.01	
Cumulative consumption	Never drinkers	18/71	1.0	
(liters/day × years)	1–47	66/156	1.7	0.9–3.3
	48–69	77/149	2.1	1.1–3.9
	70+	94/127	3.2	1.7–6.0
	p-value for trend		<0.01	
Temperature	Never drinkers	18/71	1.0	
	Warm	11/20	2.1	0.8–5.4
	Hot	191/383	2.1	1.2–3.7
	Very hot	35/27	4.9	2.2–11

OR, odds ratio adjusted for age, sex, residence, urban/rural status, education, family history of bladder cancer among first-degree relatives, body mass index, occupation, smoking status, years since quitting, number of cigarettes smoked per day, coffee drinking, tea drinking, soft drink intake, and milk intake

CI, confidence interval.

**Table 3:** Odds ratios of bladder cancer for coffee, tea and soft drinks consumption

**Coffee**				
	**Category**	**Cases/Controls**	**OR**	**95 % CI**

Drinking status	Never drinkers	135/332	1.0	
	Former drinkers	9/13	1.4	0.6–3.6
	Current drinkers	111/156	1.7	1.2–2.3
	Ever drinkers	120/169	1.6	1.2–2.3
Amount (pure coffee)	Never drinkers	135/332	1.0	
(cups/week)	1–6	22/28	1.6	0.8–3.1
	7+	15/21	2.0	0.9–4.4
	p-value for trend		0.03	
Amount (coffee with milk)	Never drinkers	135/332	1.0	
(cups/week)	1–6	70/112	1.5	1.0–2.2
	7+	24/24	1.9	1.0–3.7
	p-value for trend		0.01	
Amount (total coffee)	Never drinkers	135/332	1.0	
(cups/week)	1–6	84/133	1.5	1.1–2.2
	7+	36/36	2.1	1.2–3.6
	p-value for trend		<0.01	

**Tea**				

	**Category**	**Cases/Controls**	**OR**	**95 % CI**

Drinking status	Never drinkers	180/419	1.0	
	Former drinkers	1/2	1.5	0.1–19
	Current drinkers	74/80	2.3	1.6–3.4
	Ever drinkers	75/82	2.3	1.5–3.4
Amount (pure tea)	Never drinkers	180/419	1.0	
(cups/week)	1–6	21/11	4.4	2.0–10
	7+	20/32	1.5	0.8–2.8
	p-value for trend		0.02	
Amount (tea with milk)	Never drinkers	180/419	1.0	
(cups/week)	1–6	28/40	1.7	1.0–3.0
	7+	10/5	6.5	2.0–21
	p-value for trend		<0.01	
Amount (total tea)	Never drinkers	180/419	1.0	
(cups/week)	1–6	59/72	2.1	1.4–3.1
	7+	16/10	4.1	1.7–9.9
	p-value for trend		<0.01	

**Soft drinks**				

	**Category**	**Cases/Controls**	**OR**	**95 % CI**

Amount	Never drinkers	138/289	1.0	
(bottles/years)	1–104	65/121	1.1	0.7–1.6
	105+	52/91	1.1	0.7–1.7
	p-value for trend		0.62	

OR, odds ratio adjusted for age, sex, residence, urban/rural status, education, family history of bladder cancer among first-degree relatives, body mass index, occupation, smoking status, years since quitting, number of cigarettes smoked per day, *maté *drinking, soft drink intake, milk intake, and, when appropriate, coffee drinking and tea drinking

CI, confidence interval.

**Table 4:** Odds ratios of bladder cancer for drinking of *maté*, coffee, tea, by smoking status

**Category**	**Never smokers**			**Ever smokers**		
**Maté drinking (liters/day × years)**						

**Category**	**Cases/Controls**	**OR**	**95% CI**	**Cases/Controls**	**OR**	**95% CI**

Never drinkers	10/30	1.0		8/41	1.0	
1–47	12/48	0.95	0.32–2.86	54/108	2.51	1.06–5.95
48–69	16/38	1.35	0.46–3.95	61/109	2.97	1.25–7.03
70+	10/20	2.31	0.65–8.23	84/107	4.30	1.82–10.1
p-value for trend			0.2			<0.01

**Coffee drinking (total coffee, cups/week)**						

**Category**	**Cases/Controls**	**OR**	**95% CI**	**Cases/Controls**	**OR**	**95% CI**

Never drinkers	26/91	1.0		109/241	1.0	
1–6	17/36	2.22	0.97–5.12	67/97	1.33	0.88–2.00
7+	5/9	2.02	0.54–7.54	31/27	2.04	1.12–3.72
p-value for trend			0.08			0.01

**Tea drinking (total tea, cups/week)**						

**Category**	**Cases/Controls**	**OR**	**95% CI**	**Cases/Controls**	**OR**	**95% CI**

Never drinkers	34/111	1.0		146/308	1.0	
1–6	11/20	2.22	0.84–5.83	48/52	1.91	1.20–3.04
7+	3/5	1.52	0.27–8.59	13/5	5.90	1.97–17.6
p-value for trend			0.2			<0.01

OR, odds ratio adjusted for age, sex, residence, urban/rural status, education, family history of bladder cancer among first-degree relatives, body mass index, occupation, soft drink intake, and milk intake, and, when appropriate, *maté *drinking, coffee drinking, and tea drinking

CI, confidence interval.
